# Distinct Roles of LFA-1 and ICAM-1 on ILC2s Control Lung Infiltration, Effector Functions, and Development of Airway Hyperreactivity

**DOI:** 10.3389/fimmu.2020.542818

**Published:** 2020-10-30

**Authors:** Benjamin P. Hurrell, Emily Howard, Lauriane Galle-Treger, Doumet Georges Helou, Pedram Shafiei-Jahani, Jacob D. Painter, Omid Akbari

**Affiliations:** Department of Molecular Microbiology and Immunology, Keck School of Medicine, University of Southern California, Los Angeles, CA, United States

**Keywords:** ILC2, LFA-1, ICAM-1, trafficking, airway hyperreactivity, asthma, IL-10

## Abstract

Asthma is a heterogeneous airway inflammatory disease characterized by increased airway hyperreactivity (AHR) to specific and unspecific stimuli. Group 2 innate lymphoid cells (ILC2)s are type-2 cytokine secreting cells capable of inducing eosinophilic lung inflammation and AHR independent of adaptive immunity. Remarkably, reports show that ILC2s are increased in the blood of human asthmatics as compared to healthy donors. Nevertheless, whether ILC2 expression of adhesion molecules regulates ILC2 trafficking remains unknown. Our results show that IL-33-activated ILC2s not only express LFA-1 but also strikingly LFA-1 ligand ICAM-1. Both LFA-1^−/−^ and ICAM-1^−/−^ mice developed attenuated AHR in response to IL-33 intranasal challenge, associated with a lower airway inflammation and less lung ILC2 accumulation compared to controls. Our mixed bone marrow chimera studies however revealed that ILC2 expression of LFA-1 — but not ICAM-1 — was required for their accumulation in the inflamed lungs. Importantly, we found that LFA-1 remarkably controlled ILC2 homing to the lungs, suggesting that LFA-1 is involved in ILC2 trafficking to the lungs. Our exploratory transcriptomic analysis further revealed that ICAM-1 deficiency on ILC2s significantly affects their effector functions. While it downregulated pro-inflammatory cytokines such as *Il5*, *Il9*, *Il13*, and *Csf2*, it however notably also upregulated cytokines including *Il10* both at the transcriptomic and protein levels. These findings provide novel avenues for future investigations, as modulation of LFA-1 and/or ICAM-1 represents an unappreciated regulatory mechanism for ILC2 trafficking and cytokine production respectively, potentially serving as therapeutic target for ILC2-dependent diseases such as allergic asthma.

## Introduction

Group 2 innate lymphoid cells (ILC2)s rapidly respond to tissue-specific interleukin (IL)-33, IL-25 and thymic stromal lymphopoietin (TSLP) by releasing copious type-2 cytokines (1–4). IL-25, IL-33, and TSLP are endogenous molecules that act as danger signals recognized by the immune system. They contribute to ongoing type 2 immune responses leading to lung inflammation but can themselves directly induce cardinal features of asthma, including airway hyperreactivity (AHR) and eosinophilia, by activating ILC2s in the absence of adaptive immunity (5). It is now well appreciated that ILC2 activation is tightly regulated by complex interactions as we and others have reported that the severity of asthma symptoms is alleviated through modulation of ILC2 effector functions (3). Remarkably, reports show that ILC2s are increased in the blood of human asthmatics as compared to healthy donors, suggesting that circulating ILC2s continually contribute to the resident lung ILC2 pool (6). In line with this finding, although ILC2s are commonly known as tissue-resident cells, recent reports have shown that ILC2s migrate and accumulate in different tissues upon inflammation, including in the lungs (7–10).

ILC2 migration to inflamed tissues is an emerging feature of ILC2 biology that may be crucial to disease severity. Although leukocyte migration to the airways is the result of a complex series of events, it is in part mediated by their expression of integrins and selectins, involved in the multistep process of diapedesis (11). Integrins are formed of a beta- and an alpha-chain, with one of the most studied — leukocyte function associated antigen-1 (LFA-1) — formed of the CD18 beta-integrin chain and a distinct alpha-chain, CD11a (12). The most common ligands for LFA-1 are intercellular adhesion molecule (ICAM)-1 and ICAM-2, both constitutively expressed on endothelial cells but also on a variety of leukocytes (13). In the context of asthma, anti-LFA-1 and anti-ICAM-1 were shown long ago to be beneficial in experimental asthma through effects on CD4^+^ T-cells (14–17). In the field of innate lymphoid cells, we were the first to show that pulmonary ILC2s remarkably express both ICAM-1 and ICAM-2, suggesting that the well-known LFA-1/ICAM-1/2 molecular pair may represent a modulator of ILC2 biology (18). Prior studies have focused on the function of LFA-1 and ICAM-1/2 In the context of ILC2-dependent asthma, but the exact contributions of LFA-1, ICAM-1, and ICAM-2 in the context of ILC2 trafficking and transmigration to the lungs remains elusive (10, 19).

Herein we examined how adhesion molecules LFA-1 and LFA-1 ligands ICAM-1 and ICAM-2 are involved in ILC2 accumulation and function in the lungs. Pulmonary ILC2s constitutively express LFA-1 subunits CD18/CD11a and ICAM-2, while activation with IL-33 rapidly induces ICAM-1 expression on ILC2s. Importantly, mice genetically deficient in LFA-1 or ICAM-1 develop attenuated AHR and lung inflammation in response to IL-33 intranasal challenge. However, using a combination of approaches that includes the generation of chimeric mice, our results suggest that only the expression of LFA-1 — and not that of ICAM-1 — on ILC2s is required for their accumulation in the lungs upon inflammation. Importantly, we show that LFA-1 on ILC2s is required for their homing to the lungs, suggesting that LFA-1 is involved in ILC2 infiltration in the lungs. Although ICAM-1 was not involved in ILC2 accumulation in the lungs, using a transcriptomic approach, constitutive genetic ablation or induced blocking of ICAM-1, our exploratory results reveal that lack of ICAM-1 on activated ILC2s downregulated pro-inflammatory cytokines such as *Il5*, *Il9*, *Il13*, and *Csf2*, while it notably also upregulated cytokines including *Il10* both at the transcriptomic and protein levels.

## Material and Methods

### Mouse Experiments

Experimental protocols were approved by the USC institutional Animal Care and Use Committee (IACUC) and conducted in accordance with the USC Department of Animal Resources’ guidelines. 5-10 week old age and sex matched mice were used in the studies. C57BL/6J, BALB/cByJ, ICAM-1 deficient (B6.129S4-*Icam1* ^tm1Jcgr^/J), LFA-1 deficient (B6.129S7-*Itgal* ^tm1Bll^/J), and RAG2 deficient (C.B6(Cg)-Rag2^tm1.1Cgn^/J) mice were bred in our animal facility at the Keck School of Medicine, University of Southern California (USC).

### *In Vivo* Experiments and Tissue Preparation

When indicated, mice were challenged on three consecutive days with 0.5 µg/mouse in 50 µl of carrier-free rmIL-33 or rmIL-25 (BioLegend) diluted in PBS. On day 4, lungs were collected and processed to single cell suspensions for the indicated readout. Briefly, following transcardial perfusion with PBS 1× to clear lungs of red blood cells, collected lungs were then digested in collagenase Type IV (400 U/ml) at 37°C for 1 h and processed to single cell suspension through a 70-mm nylon cell strainer (Falcon) as described previously (20, 21). For experiments involving bone marrow cells, cells were isolated by flushing the bone with 5 ml ice cold PBS 1× from one tibia using a 25-G syringe (Becton Dickinson), red blood cells were lysed and cells used for the indicated experiment. For experiments involving peripheral blood, 2 ml of blood was collected *via* heart puncture using a 27-G needle syringe (Becton Dickinson) and kept in PBS 1% EDTA, red blood cells were lysed, and peripheral blood cells used for the indicated experiment. In experiments involving *in vivo* antibody blocking antibodies, 100 µg anti-ICAM-1 (YN1/1.7.4, BioXcell), anti-ICAM-2 (3C4, Biolegend) or corresponding isotype controls were injected through the tail-vein on days 1, 2, and 3 prior rmIL-33 intranasal challenge.

### Bone Marrow Chimera and Adoptive Transfer Experiments

CD45.1^+^ C57BL/6 host mice were first irradiated with 600 rad. The following day, bone marrow cells were isolated from the tibia of CD45.1^+^ C57BL/6 WT or CD45.2^+^ KO (LFA or ICAM-1) donors as indicated above, and a CD45.1 to CD45.2 1:1 mix was prepared prior to adoptive transfer of 5 × 10^6^ cells to host mice through the tail-vein. After transfer, mice were maintained in our pathogen-free facility for 4-6 weeks, and the chimeric blood ratio was measured 2 weeks after transfer and a day prior start of experiment. For other adoptive transfer experiments, activated lung CD45.2^+^ ILC2s (either C57BL/6 or LFA-1^−/−^) were FACS-sorted as described below. At the indicated time, C57BL/6 CD45.1^+^ host mice were adoptively transferred with 10 × 10^3^ FACS-sorted CD45.2^+^ activated lung ILC2 (C57BL/6 or LFA-1^−/−^) by tail-vein injection.

### Murine ILC2 and *In Vitro* Culture

Murine ILC2s were FACS-sorted to a purity of >95% on a FACSARIA III system. ILC2s were purified from the lungs of naïve mice, whereas aILC2s from mice challenged on three consecutive days with 0.5 µg/mouse in 50 µl of carrier-free rmIL-33 (BioLegend). ILC2s were gated as lineage (CD3ε, CD4, CD5, CD45R, Gr-1, CD11c, CD11b, Ter119, TCRγδ, TCRβ, and FCεRIα) negative CD45^+^, ST2^+^, CD127^+^ cells. Isolated ILC2s were cultured at 37°C (5 × 10^4^/ml) with rmIL-2 (10 ng/ml) and rmIL-7 (10 ng/ml) purchased from BioLegend in complete RPMi (cRPMi). For cRPMi, RPMI (Gibco) was supplemented with 10% heat-inactivated FBS (Omega Scientific), 100 units/ml penicillin and 100 mg/ml streptomycin (GenClone). When stated, nILC2s were in addition activated with 50 ng/ml rmIL-33 and CD18, CD11a, ICAM-1, and ICAM-2 expressions were assessed by flow cytometry at the indicated times. In ICAM-1 blocking experiments, 10 µg/ml anti-ICAM-1 (YN1/1.7.4, Thermofisher) or corresponding isotype were added to culture.

### Flow Cytometry

The following murine antibodies were used: biotinylated anti-mouse lineage CD3ε (145-2C11), CD4 (GK1.5), CD5 (53-7.3), TCRβ (H57-597), CD45R (RA3-6B2), Gr-1 (RB6-8C5), CD11c (N418), CD11b (M1/70), Ter119 (TER-119), FcεRIα (MAR-1), Streptavidin-FITC, PE-Cy7 anti-mouse CD127 (A7R34), APCCy7 anti-mouse CD45 (30-F11), PECy7 anti-mouse CD45 (30-F11), APCCy7 anti-mouse CD11c (N418), FITC anti-mouse CD19 (6D5), APC anti-mouse Gr-1 (RB6-8C5), PerCPCy5.5 anti-mouse CD3 (17A2), Alexa Fluor 647 anti-mouse CD102 (3C4), FITC anti-mouse CD102 (3C4), BV510 anti-mouse CD45.1 (A20), APC anti-mouse CD45.2 (104), APC anti-mouse KLRG1 (2F1), APC anti-mouse CD25 (3C7) were purchased from BioLegend. PE anti-mouse CD18 (C71/16), APC anti-mouse CD11a (2D7), BV510 anti-mouse CD54 (3E2), PE anti-mouse SiglecF (E50-2440) were purchased from BD Biosciences. APC anti-mouse CD90.2 (53-2.1), TCR-γδ (eBioGL3), PerCP-eFluor710 anti-mouse ST2 (RMST2-2), eFluor450 anti-mouse CD11b (M1/70) were purchased from Thermofisher. Intranuclear staining was performed using the Foxp3 Transcription Factor Staining Kit (Thermofisher) according to the manufacturer’s instructions and APC anti-mouse Ki67 (SolA15, Thermofisher) or PE anti-mouse/human GATA3 (TWAJ, Thermofisher) were used. Intracellular staining was performed using the BD Biosciences Cytofix/Cytoperm kit. When indicated, cells producing cytokines was measured following 4 h *in vitro* stimulation with 50 µg/ml PMA, 500 µg/ml ionomycin (both Sigma) and 1 µg/ml Golgi plug (BD Biosciences) using the BD Cytofix/Cytoperm Plus staining kit (BD Biosciences). PE anti-mouse IL-10 (JES5-16E3, BD Biosciences), eFluor 450 anti-mouse IL-13 (eBio13A, Thermofisher), PE anti-mouse/human IL-5 (TRFK5, Biolegend) were used. For apoptosis staining, PE Annexin V (Thermofisher) and DAPI (Sigma) were used according to the manufacturer’s instructions. Live/dead fixable violet cell stain kit was used to exclude dead cells, used according to the manufacturer’s instructions (Thermofisher) and CountBright absolute counting beads (Thermofisher) to calculate absolute cell numbers when indicated. Stained cells were analyzed on FACSCanto II and/or FACSARIA III systems and the data was analyzed with FlowJo version 9 software.

### Measurement of Lung Function

On day 4 following rmIL-33 intranasal challenge, lung function was measured using the FinePointe RC system (Buxco Research Systems) as described previously (20, 22, 23). Briefly, mice were surgically tracheotomized under deep anesthesia and placed on the mechanically ventilated system where increasing doses of methacholine (acetyl-b-methylcholine chloride (Sigma) are sequentially nebulized. Methacholine is a bronchoconstrictor inducing airway contraction and was nebulized at various doses in 10 µl, ranging from 0 to 40 mg/ml. For each dose, lung resistance was measured and computed over a period of 3 min.

### Cytokine Measurements and Collection of BAL Fluid

The amounts of cytokines in culture supernatants were measured by ELISA as described previously (24). Murine IL-5, IL-13, and IL-10 ELISA kits were all purchased from Thermofisher and used according to the manufacturer’s instructions. BAL fluid was collected as previously described (20). Briefly, the lungs of tracheotomized mice were washed three times with 1 ml PBS 1× to collect cells. Following red blood cell lysis, cells were stained for flow cytometry analysis. Eosinophils were gated as CD45^+^ CD11c^−^ SiglecF^+^ single cells.

### RNA Sequencing and Data Analysis

Transcriptomic analysis was performed as described previously (20, 25). Briefly, FACS-sorted pulmonary ILC2s were recovered, directly lysed in RLT buffer (Qiagen), and RNA was extracted using the MicroRNeasy kit (Qiagen). For each sample, a total of 10 ng of RNA was used to generate cDNA (SMARTer Ultra Low Input RNA v3 kit, Clontech) for library preparation. Samples were then amplified and sequenced on a NextSeq 500 system (Illumina) where on average 30 million reads were generated from each sample. Raw reads were then further processed on Partek Genomics Suite software, version 7.0 Copyright ^©^; Partek Inc. Briefly, raw reads were aligned by STAR — 2.6.1 d with mouse reference index mm10 and GENECODE M20 annotations. Aligned reads were further quantified and normalized using the upper quartile method and differential analysis by GSA. Transcripts showing an average normalized count below 1 were removed from the analysis, as were genes showing cumulative normalized counts below 10.

### Statistical Analysis

Experiments were repeated at least three times (n = 4–8 each) and data are shown as the representative of >2 independent experiments, except for the RNAseq performed in **Figure 6**. Non-parametric tests were used: Mann-Whitney U tests were used to compare the differences between two groups, except for multi-group comparisons where Kruskal Wallis tests were used. All tests were performed using Prism Software (GraphPad Software Inc.). The degree of significance was indicated as: *p < 0.05, **p < 0.01, ***p < 0.001.

## Results

### Pulmonary ILC2s Express LFA-1, ICAM-1, and ICAM-2

**Figure 1:**
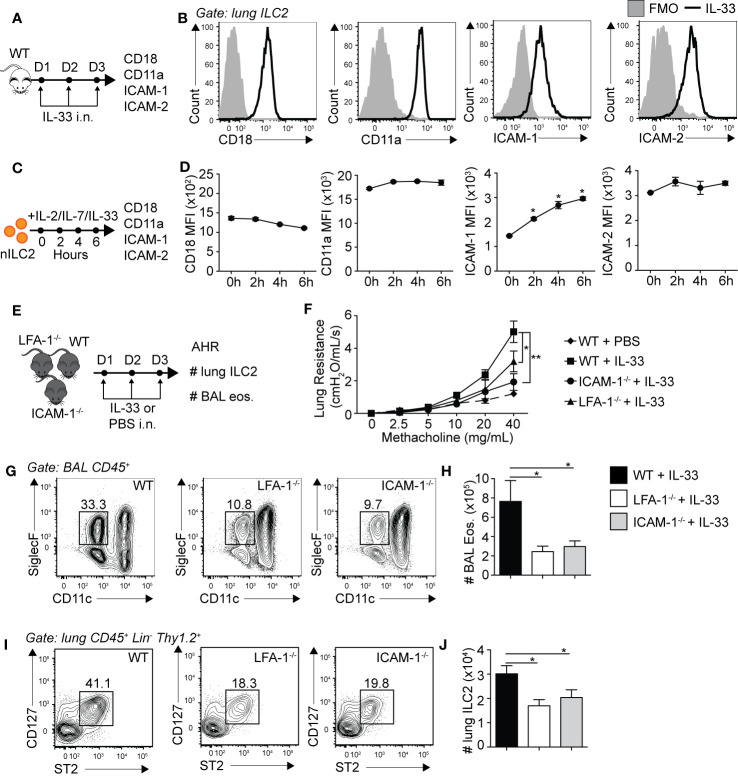
Pulmonary ILC2s express LFA-1, ICAM-1 and ICAM-2 as both ICAM-1^−/−^ and LFA-1^−/−^ mice develop attenuated ILC2-dependent AHR. **(A)** A cohort of BALB/cByJ mice were challenged intranasally on days 1-3 with 0.5 µg rmIL-33 and on day 4 lungs were recovered, processed to single cell suspensions and the expression of CD18, CD11a, ICAM-1, and ICAM-2 on pulmonary CD45^+^ Lin^−^ Thy1.2^+^ ST2^+^ CD127^+^ ILC2s was analyzed by flow cytometry. **(B)** Representative flow cytometry plots of CD18, CD11a, ICAM-1, and ICAM-2 expressions on pulmonary ILC2s following challenge with rmIL-33. Black line: Corresponding marker, Solid Gray: Full Minus One (FMO) staining control. **(C)** Lungs from a cohort of naïve BALB/cByJ mice were recovered, processed to single cell suspensions and pulmonary naïve ILC2s (nILC2)s were FACS-sorted to a purity of >95%. Pure nILC2s were then cultured (50 × 10^4^/ml) *in vitro* at 37°C in the presence of rmIL-2 (10 ng/ml), rmIL-7 (10 ng/ml), and rmIL-33 (50 ng/ml) and analyzed for the expression of CD18, CD11a, ICAM-1, and ICAM-2 at the indicated times by flow cytometry. **(D)** Mean Fluorescence Intensity (MFI) of CD18, CD11a, ICAM-1 and ICAM-2 on cultured nILC2s at the indicated times after rmIL-33 stimulation *in vitro*, presented as MFI ± SEM. **(E)** Separate cohorts of C57BL/6 (WT), LFA-1^−/−^ and ICAM-1^−/−^ mice were challenged intranasally on days 1 to 3 with 0.5 µg rmIL-33. On day 4, airway hyperreactivity (AHR) was analyzed in each cohort as a measure of lung resistance by sequentially nebulizing increasing doses of bronchoconstrictor methacholine in anesthetized/tracheotomized mice on the FinePointe RC system (Buxco Research Systems). Additionally, the numbers ILC2s (CD45^+^ Lin^−^ Thy1.2^+^ ST2^+^ CD127^+^) in the lungs, and eosinophils (CD45^+^ SiglecF^+^ CD11c^−^) in the bronchoalveolar lavage (BAL) fluid were analyzed by flow cytometry. **(F)** Lung resistance in each cohort of mice in response to increasing doses of bronchoconstrictor methacholine. The dashed line represents WT mice challenged intranasally with PBS as a baseline control for the measure of AHR. **(G)** Representative flow cytometry plots of BAL eosinophils in each cohort and **(H)** corresponding quantitation presented as the mean number of BAL eosinophils ± SEM. **(I)** Representative flow cytometry plots of pulmonary ILC2s in each cohort and **(J)** corresponding quantitation presented as the mean number of lung ILC2s ± SEM. Data are representative of three individual experiments with n = 5. *p < 0.05, **p < 0.01.

Leukocyte trafficking to inflamed tissues such as the lungs is in part controlled by the expression of adhesion molecules, with the LFA-1/ICAM-1/2 molecular interaction crucial in controlling immune cell diapedesis. We therefore first challenged WT mice for three consecutive days with rmIL-33 intranasally (i.n.) and measured the levels of expression of ICAM-1, ICAM-2 and LFA-1 subunits CD18/CD11a on lung ILC2s by flow cytometry on day 4 (**Figure 1A**). ILC2s were gated as live CD45^+^, Lineage^-^, Thy1.2^+^, CD127^+^ and ST2^+^ cells. An extensive murine activated ILC2 (aILC2) gating strategy can be found in **Supplementary Figure 1**. We found that pulmonary activated ILC2s highly express both LFA-1 subunits CD18 and CD11a, but surprisingly also corresponding ligands ICAM-1 and ICAM-2 (**Figure 1B**). We next assessed expression dynamics of these adhesion molecules on lung ILC2s. We therefore FACS-sorted naïve lung ILC2s (nILC2)s from a cohort of naïve WT mice and cultured them *in vitro* in the presence of rmIL-33 to measure the expression of CD18, CD11a, ICAM-1, and ICAM-2 on ILC2s over time by flow cytometry (**Figure 1C**). We found that CD18 and CD11a as well as ICAM-2 were expressed by lung nILC2 but not induced by rmIL-33 *in vitro* (**Figure 1D**). Remarkably however, pulmonary nILC2 expressed low levels of ICAM-1, which were rapidly induced within as fast as 2 h by rmIL-33 *in vitro*. nILC2s incubated without rmIL-33 did not induce ICAM-1 expression over time, suggesting that our observations are specific to IL-33 stimulation (data not shown). Furthermore, the upregulation of ICAM-1 upon rmIL-33 stimulation on ILC2s as well as expression of ICAM-2 and LFA-1 subunits are independent of the genetic background, as we observed similar results in both BALB/c and C57BL/6 mice (**Supplementary Figures 3A, B**). Interestingly, IL-25 also induced ICAM-1 expression on ILC2s, albeit at a lower level as compared to rmIL-33 (**Supplementary Figures 3A, B**). Together, our findings suggest that LFA-1 and ICAM-2 are constitutively expressed by naïve and activated pulmonary ILC2s. Furthermore, naïve ILC2s express low levels of ICAM-1 that are rapidly increased following IL-33 stimulation.

### LFA-1, ICAM-1 but Not ICAM-2 Are Required for ILC2-Dependent AHR

We next assessed the biological relevance of LFA-1, ICAM-1 and ICAM-2 expressions on the development of ILC2-dependent AHR and airway inflammation. AHR is a powerful tool allowing the measure of airway resistance, a cardinal feature of asthma, as described previously (18–20, 25). Briefly, mice were surgically tracheotomized under deep anesthesia and placed on a mechanically ventilated system where increasing doses of methacholine are sequentially nebulized. Methacholine is a bronchoconstrictor inducing airway contraction and was nebulized at various doses ranging from 0 to 40 mg/ml. For each dose, lung resistance was measured and computed. We therefore treated cohorts of WT, LFA-1^−/−^ and ICAM-1^−/−^ mice with rmIL-33 or PBS i.n. for three consecutive days and measured AHR on day 4 (**Figure 1E**). LFA-1^−/−^ mice constitutively lack both CD18 and CD11a, whereas ICAM-1^−/−^ mice constitutively lack ICAM-1, without affecting ICAM-2 expression (**Supplementary Figure 2**). As expected, we first observed that WT mice challenged with rmIL-33 induced significantly higher lung resistance as compared to mice challenged with PBS (**Figure 1F**). However, LFA-1^−/−^ and ICAM-1^−/−^ mice developed significantly less lung resistance as compared to WT controls in response to rmIL-33 challenge, suggesting that the expression of LFA-1 or ICAM-1 are required for efficient development of AHR in response to IL-33 (**Figure 1F**). Furthermore, airway inflammation was lower in both LFA-1^−/−^ and ICAM-1^−/−^ mice compared to WT mice, as the decrease in AHR was associated with decreased numbers of eosinophils in the bronchoalveolar (BAL) fluid (**Figures 1G, H**). Since ILC2s play a central role in the development of IL-33-induced AHR, we next measured the numbers of pulmonary ILC2s in each cohort. We found that both LFA-1^−/−^ and — as reported previously (19) — ICAM-1^−/−^ mice harbored significantly less lung ILC2s compared to WT mice (**Figures 1I, J**). Importantly, we also assessed the requirement of ICAM-2 expression on airway inflammation using an anti-ICAM-2 blocking strategy (**Supplementary Figure 5**). Strikingly, blocking ICAM-2 neither affected airway inflammation nor the number of pulmonary ILC2s following intranasal challenge with rmIL-33 on three consecutive days. In addition, we did not observe an increase in ICAM-2 expression in ICAM-1^−/−^ activated lung ILC2s, suggesting that ICAM-1 and ICAM-2 are not functionally redundant in this context (**Supplementary Figures 2D–F**). Together, our findings reveal that LFA-1 and ICAM-1 — but not ICAM-2 — are required for ILC2 accumulation in the lungs and development of ILC2-dependent AHR.

### LFA-1 on ILC2s Is Required for Accumulation in the Lungs

**Figure 2:**
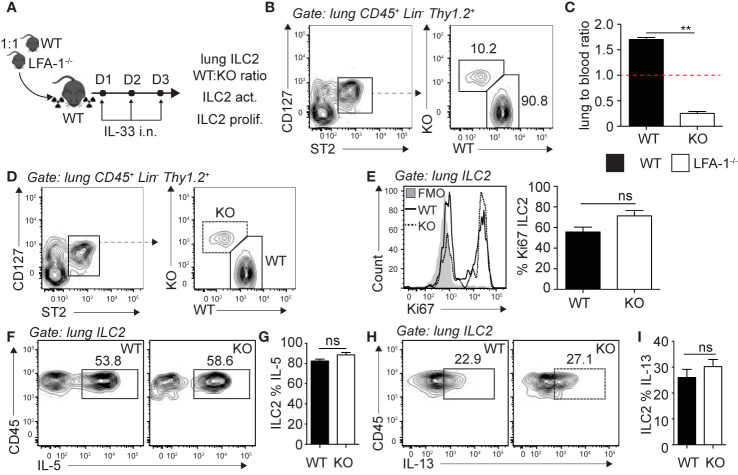
LFA-1 on ILC2s is required for ILC2 accumulation in the lungs. **(A)** 5 × 10^6^ 1:1 mix of WT (CD45.1): LFA-1^−/−^ (CD45.2) bone marrow cells were intravenously injected through the tail-vein to lethally irradiated WT (CD45.1) host mice. 4 weeks after transfer, mice were challenged intranasally on days 1-3 with 0.5 µg rmIL-33. On day 4, lungs were recovered, processed to single cell suspensions and analyzed for the presence of pulmonary ILC2s (CD45^+^ Lin^−^ Thy1.2^+^ ST2^+^ CD127^+^). We further measured the expression of CD45.1 (WT) or CD45.2 (LFA-1^−/−^) within total lung ILC2s by flow cytometry **(B, C)**, as well as the expression of Ki67 as a measure of proliferation **(D, E)** and intracellular IL-5 and IL-13 as a measure of activation **(F–I)** by flow cytometry in both CD45.1 (WT) and CD45.2 (LFA1^−/−^) pulmonary ILC2s. **(B)** Representative flow cytometry plots of total lung ILC2s expressing either CD45.1 (WT) or CD45.2 (LFA-1^−/−^) and **(C)** corresponding quantitation presented as the ratio of CD45.1 (WT) and CD45.2 (LFA-1^−/−^) ILC2s within total lung ILC2s, normalized to the peripheral blood CD45.1:CD45.2 ratio of each individual mice ± SEM. Red dotted line represents the ratio of 1. **(D)** Representative flow cytometry plots of CD45.1^+^ (WT) and CD45.2^+^ (LFA-1^−/−^) expression within total lung ILC2s. **(E)** Representative flow cytometry plots depicting the frequency of Ki67 expression within CD45.1^+^ and CD45.2^+^ lung ILC2 populations and corresponding quantitation presented as the mean frequency of Ki67 expression within CD45.1^+^ and CD45.2^+^ lung ILC2s ± SEM. **(F–I)** After processing to single cell suspension, lung cells were further stimulated with PMA, ionomycin and Brefeldin A for 4 h at 37°C prior staining for intracellular cytokine markers. Representative flow cytometry plots of IL-5 **(F)** and IL-13 **(H)** expression within CD45.1^+^ and CD45.2^+^ lung ILC2 populations and corresponding quantitation **(G, I)** presented as mean frequency of cytokine expression within CD45.1^+^ and CD45.2^+^ lung ILC2s ± SEM. Data are representative of 3 individual experiments with n = 5. **p < 0.005, ns, non-significant.

Following the observation that pulmonary ILC2s are decreased in the constitutive absence of LFA-1, we next investigated the functional requirement of LFA-1 on ILC2s for their accumulation in the lungs during inflammation. To do so, we generated mixed bone marrow (BM) chimera experiments using the WT (CD45.1) and LFA-1^−/−^ (CD45.2) mice models that allow tracking of immune cells within tissues based on their expression of the CD45 isoform. Using this approach, we therefore directly assessed the function of LFA-1 expressed on ILC2s for their accumulation in the lungs. Lethally irradiated CD45.1 (host) mice were reconstituted with a mixture of WT (CD45.1) and LFA-1^−/−^ (CD45.2) BM hematopoietic cells (**Figure 2A**). There was an average peripheral blood CD45.1:CD45.2 ratio of 50:50 4 weeks following reconstitution (data not shown). We next further challenged chimeric mice on three consecutive days with rmIL-33 i.n. and measured the lung ILC2 CD45.1:CD45.2 ratio on day 4 (**Figure 2A** **).** Strikingly, we found that WT (CD45.1) ILC2s represented an average 90% of total lung ILC2s, whereas LFA-1^−/−^ (CD45.2) ILC2s only accounted for 10% of total lung ILC2s (**Figure 2B**). When we normalized the CD45.1:CD45.2 ratio of lung ILC2s to that of the peripheral blood of individual mice, we found a significant shift towards WT ILC2s, suggesting that the expression of LFA-1 on ILC2s is required for their accumulation in the lungs (**Figure 2C**). We next measured the functional requirement of LFA-1 on pulmonary ILC2s for their proliferation (**Figures 2D, E**) and cytokine secretion (**Figures 2F–I**), as both can affect airway inflammation by modulating ILC2 numbers and activation, respectively. Our results suggest that LFA-1 on lung ILC2s does not affect their proliferation, as both WT (CD45.1) and LFA-1^−/−^ (CD45.2) lung ILC2s expressed similar levels of nuclear cell proliferation marker Ki67 (**Figures 2D, E**). Similarly, the expression of LFA-1 on ILC2s did not appear to modulate intracellular IL-5 and IL-13 secretion, as we did not observe differences in the frequencies of WT (CD45.1) or LFA-1^−/−^ (CD45.2) ILC2s expressing these cytokines (**Figures 2F–I**). Together, our results suggest that LFA-1 on ILC2s controls their accumulation in the lungs during inflammation.

### ILC2 Expression of LFA-1 Is Not Required for ILC2 Homeostasis in the Lungs

**Figure 3:**
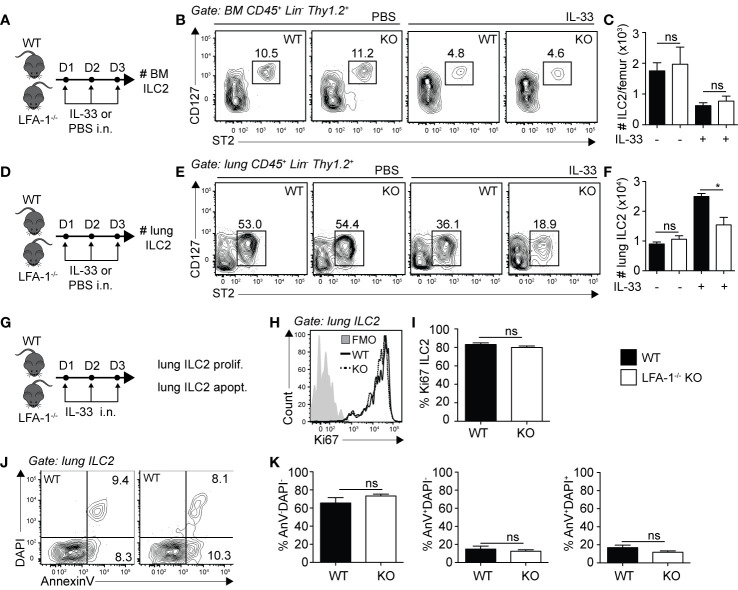
LFA-1 on ILC2s is not required for ILC2 homeostasis in the lungs. **(A, D)** Cohorts of C57BL/6 (WT) and LFA-1^−/−^ mice were challenged intranasally on days 1-3 with either PBS or 0.5 µg rmIL-33. On day 4, lungs were recovered as well as the left femur where bone marrow cells were flushed in PBS. Both tissues were processed to single cell suspensions, and the presence of CD45^+^ Lin^−^ Thy1.2^+^ ST2^+^ CD127^+^ ILC2s was analyzed by flow cytometry in both the bone marrow **(A–C)** and the lungs **(D, E)**. **(B)** Representative flow cytometry plots of PBS-treated (left) and IL-33-treated (right) bone marrow ILC2s in both WT and LFA-1^−/−^ ILC2s. Corresponding quantitation in **(C)** is shown presented as the mean number of bone marrow ILC2s ± SEM. **(E)** Representative flow cytometry plots of PBS-treated (left) and IL-33-treated (right) lung ILC2s in both WT and LFA-1^−/−^ ILC2s. Corresponding quantitation in **(F)** is shown presented as the mean number of lung ILC2s ± SEM. **(G)** Cohorts of C57BL/6 (WT) and LFA-1^−/−^ mice were challenged intranasally on days 1 to 3 with 0.5 µg rmIL-33 and on day 4 lungs were recovered and processed to single cells suspensions. Lung ILC2s were then analyzed for proliferation (%Ki67^+^) **(H, I)** and apoptosis/survival (% AnnexinV^+^ DAPI^+^) **(J, K)** by flow cytometry. **(H)** Representative flow cytometry plots depicting the frequency of Ki67 expression within lung ILC2 populations from WT and LFA-1^−/−^ mice and **(I)** corresponding quantitation presented as mean frequency of Ki67 expression within lung ILC2s ± SEM. **(J)** Representative flow cytometry plots depicting the frequencies of AnnexinV^-^DAPI^−^ (live), AnnexinV^+^DAPI^−^ (early apoptotic) and AnnexinV^+^DAPI^+^ (late apoptotic/necrotic) lung ILC2s from WT and LFA-1^−/−^ mice and **(K)** corresponding quantitation presented as the mean frequencies of AnnexinV^-^DAPI^−^, AnnexinV^+^DAPI^−^ and AnnexinV^+^DAPI^+^ within lung ILC2s ± SEM. Data are representative of three individual experiments with n = 5. *p < 0.05, ns, non-significant.

Differences in cell numbers found in inflamed tissues such as the lungs can be the result of multiple factors that include defects in cell development, egress from the bone marrow, proliferation or survival. We therefore next focused on the role of LFA-1 on ILC2 development and lung homeostasis. We first measured the numbers of ILC2s in the bone marrow of WT and LFA-1^−/−^ mice, both at steady state and following intranasal challenge with rmIL-33 (**Figure 3A**). Our results suggest that there is no effect of LFA-1 on ILC2 development, as evidenced by the comparable numbers of BM ILC2s in both WT and LFA-1^−/−^ mice at steady state (**Figure 3B**, left panel, and **Figure 3C**). Importantly, we found that both WT and LFA-1^−/−^ mice harbored similarly decreased numbers of BM ILC2s following challenge with rmIL-33, indicating that ILC2 egressed from the bone marrow independently of LFA-1 (**Figures 3B, C**). In the same cohorts, we next measured the numbers of pulmonary ILC2s (**Figure 3D**). In line with our previous observations in the bone marrow, LFA-1 did not affect ILC2 numbers at steady state in the lungs (**Figures 3E, F**). Remarkably however, a significant increased number of ILC2s accumulated in the lungs in WT compared to LFA-1^−/−^ mice in response to i.n. challenge with rmIL-33 (**Figures 3E, F**). We next measured ILC2 proliferation and survival in LFA-1^−/−^ lung ILC2s, as both can affect total lung ILC2 numbers in the context of inflammation. We therefore challenged WT and LFA-1^−/−^ mice with rmIL-33 i.n. and measured nuclear protein Ki67 expression within lung ILC2s as a measure of proliferation (**Figure 3G**). Similar to our findings shown in **Figure 2E**, LFA-1 did not appear to affect ILC2 proliferation (**Figures 3H, I**). In the same line, we measured the frequencies of Annexin V and DAPI in pulmonary ILC2s as a measure of cell survival and apoptotic rate (**Figure 3G**). There was no significant induction of ILC2 apoptosis in LFA-1^−/−^ ILC2s compared to controls in the inflamed lungs, suggesting that LFA-1 does not affect pulmonary ILC2 survival (**Figures 3J, K**). Together, our findings reveal that LFA-1 does not control ILC2 development or retention in the bone marrow. Furthermore, LFA-1 did not affect ILC2 survival or proliferation in the activated lungs.

### LFA-1 on ILC2s Controls ILC2 Homing to the Activated Lungs

**Figure 4:**
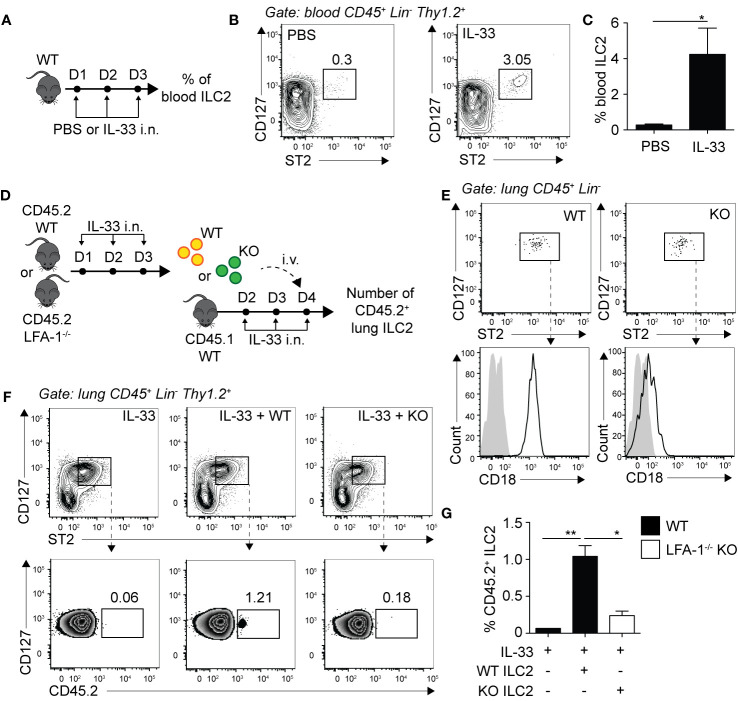
LFA-1 on ILC2s controls ILC2 homing to the activated lungs. **(A)** C57BL/6 mice were challenged intranasally on days 1 to 3 with 0.5 µg rmIL-33 or PBS and on day 4 the frequency of CD45^+^ Lin^−^ Thy1.2^+^ ST2^+^ CD127^+^ ILC2s in the peripheral blood was analyzed by flow cytometry. **(B)** Representative flow cytometry plots of peripheral blood ILC2s and **(C)** corresponding quantitation presented as the mean frequency ± SEM. **(D)** Cohorts of C57BL/6 (CD45.2) and LFA-1^−/−^ (CD45.2) mice were challenged intranasally on days 1, 2, and 3 with 0.5 µg rmIL-33. On day 4, lungs were recovered, processed to single cell suspensions and pulmonary activated ILC2s (aILC2)s were FACS-sorted to a purity of >95%. Concurrently, cohorts of C57BL/6 (CD45.1) host mice were challenged intranasally on days 2, 3 and 4 with 0.5 µg rmIL-33. On day 4, host mice were adoptively transferred with 10 × 10^3^ FACS-sorted CD45.2^+^ aILC2 (WT or LFA-1^−/−^) by tail-vein injection in the corresponding cohorts. On day 5, lungs were recovered, processed to single cell suspensions and analyzed by flow cytometry for the presence of pulmonary CD45.2^+^ ILC2s. **(E)** Flow cytometry analysis of FACS-sorted pulmonary C57BL/6 and LFA-1^−/−^ ILC2s and corresponding expression of LFA-1 subunit CD18 prior tail-vein injection to host mice. **(F)** Representative flow cytometry plots of total pulmonary ILC2s in the corresponding cohorts (upper) and frequencies of CD45.2^+^ adoptively transferred ILC2s within each population (lower). **(G)** Corresponding quantitation presented as the mean frequencies of CD45.2^+^ within total lung ILC2s ± SEM. Data are representative of 3 individual experiments with n = 5–10. *p < 0.05, **p < 0.01.

We next assessed whether LFA-1 controlled ILC2 infiltration to the lungs by focusing on ILC2 homing to the inflamed lungs. We challenged WT mice with either PBS or rmIL-33 on three consecutive days i.n. and quantified the frequencies of peripheral blood ILC2s on day 4 by flow cytometry (**Figure 4A**). Compared to the controls, we found that the peripheral blood of mice challenged with rmIL-33 harbored on average 10 times more ILC2s, with the frequency of CD45^+^ Lineage^-^ ILC2s increasing from 0.3% to 3.05% (**Figures 4B, C**). An increased proportion of ILC2s is therefore found in the peripheral blood during lung inflammation, likely egressing from the BM and/or tissues including the inflamed lungs. In order to assess the role of LFA-1 in ILC2 homing during inflammation, we next designed adoptive transfer experiments using the CD45.1/CD45.2 mice models, allowing for donor/recipient cells to be distinguished based on their expression of the CD45.1 or CD45.2 isoforms by flow cytometry (**Figure 4D** **).** We therefore challenged separate cohorts of C57BL/6 (WT) and LFA-1^−/−^ (both CD45.2^+^) donor mice with rmIL-33 on days 1-3, and on day 4 FACS-sorted lung activated ILC2s (aILC2s) to a high purity (**Figure 4E**). As a control, we confirmed that sorted aILC2s from LFA-1^−/−^ mice lacked LFA-1 subunit CD18 (**Figure 4E**). Concurrently, we treated cohorts of C57BL/6 CD45.1^+^ recipient mice with rmIL-33 on days 2-4, and on day 4 adoptively transferred FACS-sorted WT or LFA-1^−/−^ aILC2s to the corresponding cohorts. On day 5 and within 24 h, we found that adoptively transferred WT aILC2s (**Figure 4F** ****, center) efficiently infiltrated the lungs as compared to un-injected controls (**Figure 4F**, left**)**. However strikingly, adoptively transferred LFA-1^−/−^ aILC2s significantly failed to home back to the lungs compared to WT controls (**Figures 4F**, right and **G**). Interestingly, WT aILC2s also infiltrated the lungs of naïve mice within 24 h, albeit at a lower level (**Supplementary Figure 3C**). These results therefore suggest that while WT ILC2s efficiently home back to the lungs and contribute to the lung ILC2 pool, the absence of LFA-1 on aILC2s significantly affects their trafficking to the activated lungs.

### ICAM-1 on ILC2s Is Not Required for ILC2 Accumulation in the Lungs

**Figure 5:**
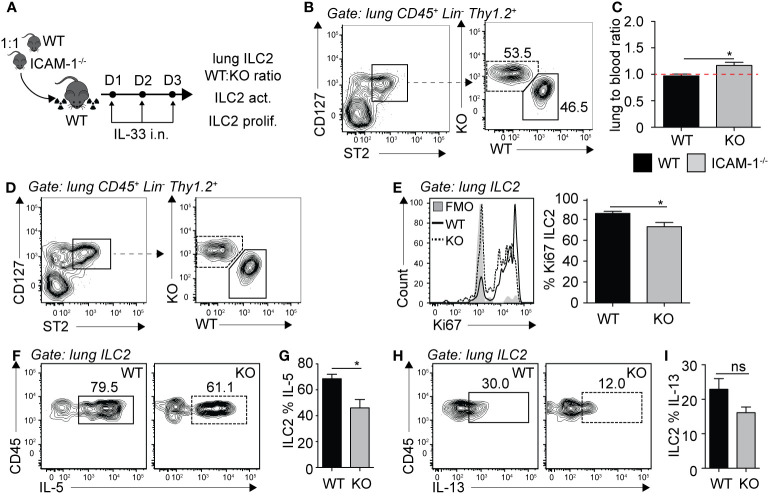
ICAM-1 on ILC2s is not required for ILC2 accumulation in the lungs. **(A)** 5 × 10^6^ 1:1 mix of WT (CD45.1): ICAM-1^−/−^ (CD45.2) bone marrow cells were intravenously injected through the tail-vein to lethally irradiated WT (CD45.1) host mice. 4 weeks after transfer, mice were challenged intranasally on days 1-3 with 0.5 µg rmIL-33. On day 4, lungs were recovered, processed to single cell suspensions and analyzed for the presence of pulmonary ILC2s (CD45^+^ Lin^−^ Thy1.2^+^ ST2^+^ CD127^+^). We further measured the expression of CD45.1 (WT) or CD45.2 (ICAM -1^−/−^) within total lung ILC2s by flow cytometry **(B, C)**, as well as the expression of Ki67 as a measure of proliferation **(D, E)** and intracellular IL-5 and IL-13 as a measure of activation **(F–I)** by flow cytometry in both CD45.1 (WT) and CD45.2 (ICAM-1^−/−^) pulmonary ILC2s. **(B)** Representative flow cytometry plots of total lung ILC2s expressing either CD45.1 (WT) or CD45.2 (ICAM -1^−/−^) and **(C)** corresponding quantitation presented as the ratio of CD45.1 (WT) and CD45.2 (ICAM -1^−/−^) ILC2s within total lung ILC2s, normalized to the peripheral blood CD45.1:CD45.2 ratio of each individual mice ± SEM. Red dotted line represents the ratio of 1. **(D)** Representative flow cytometry plots of CD45.1^+^ (WT) and CD45.2^+^ (ICAM-1^−/−^) expression within total lung ILC2s. **(E)** Representative flow cytometry plots depicting the frequency of Ki67 expression within CD45.1^+^ and CD45.2^+^ lung ILC2 populations and corresponding quantitation presented as mean frequency of Ki67 expression within CD45.1^+^ and CD45.2^+^ lung ILC2s ± SEM. **(F-I)** After processing to single cell suspension, lung cells were further stimulated with PMA, ionomycin and Brefeldin A for 4 h at 37°C prior staining for intracellular cytokine markers. Representative flow cytometry plots of IL-5 **(F)** and IL-13 **(H)** expression within CD45.1^+^ and CD45.2^+^ lung ILC2 populations and corresponding quantitation **(G, I)** presented as mean frequency of cytokine expression within CD45.1^+^ and CD45.2^+^ lung ILC2s ± SEM. Data are representative of 3 individual experiments with n = 5. *p < 0.05, ns, non-significant.

We next sought to investigate the requirement of ILC2 expression of ICAM-1 on ILC2 accumulation in the lungs. Importantly, ICAM-1 is constitutively expressed by various cells and in particular the vasculature, shown in various models to bind to infiltrating cells expressing LFA-1 (27–29). Similar to our approach in **Figure 2**, we generated mixed bone marrow chimera experiments using the WT (CD45.1) and ICAM-1^−/−^ (CD45.2) mice models. There was an average peripheral blood CD45.1:CD45.2 ratio of 60:40 4 weeks following reconstitution (data not shown). We next further challenged chimeric mice on three consecutive days with rmIL-33 i.n. and measured the lung ILC2 CD45.1:CD45.2 ratio on day 4 (**Figure 5A**). When we normalized the CD45.1:CD45.2 ratio of lung ILC2s to that of peripheral blood of each individual mice, our results suggest that ICAM-1^−/−^ ILC2s accumulated as efficiently as WT ILC2s in the lungs, if not better (**Figures 5B, C**). As we did in the context of LFA-1-expressing ILC2s, we next measured the functional requirement of ICAM-1 for ILC2 proliferation (**Figures 5D, E**), and cytokine production (**Figures 5F–I**). Strikingly, we found that ICAM-1^−/−^ ILC2s proliferated at a significantly lower rate compared to controls, as observed by the lower expression of nuclear proliferation marker Ki67 within ICAM-1^−/−^ (CD45.2) lung ILC2s compared to WT controls (CD45.1) (**Figure 5E**). As reported previously, we observed lower IL-5 (**Figures 5F, G**) and IL-13 (**Figures 5H, I**) expression in ICAM-1^−/−^ (CD45.2) ILC2s compared to WT controls (CD45.1), as ICAM-1 appeared to modulate intracellular cytokine secretion within pulmonary ILC2s (19). Together, our results suggest that — unlike in the context of LFA-1 on ILC2s — ICAM-1 on ILC2s does not control their accumulation to the lungs. However, we found a significant effect of ICAM-1 on ILC2 proliferation and activation.

### ICAM-1 on ILC2s Controls ILC2 Effector Functions

**Figure 6:**
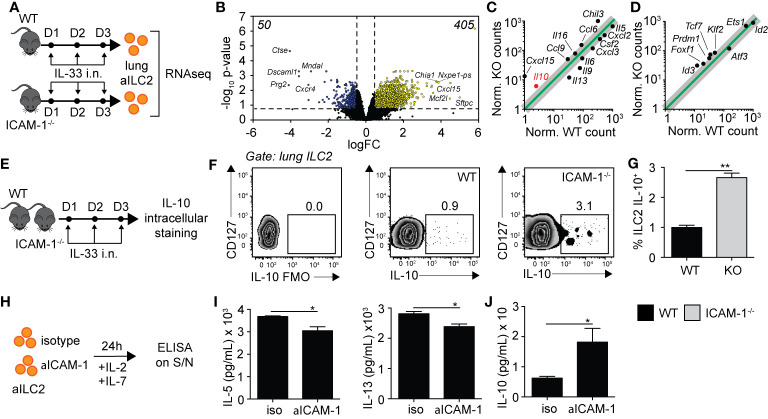
ICAM-1 on ILC2s controls ILC2 effector functions. **(A)** Cohorts of C57BL/6 (WT) and ICAM-1^−/−^ mice were challenged intranasally on days 1 to 3 with 0.5 µg rmIL-33. On day 4, lungs were recovered, processed to single cell suspensions, and pulmonary activated ILC2s (gated as CD45^+^ Lin^−^ ST2^+^ CD127^+^) were FACS-sorted to a purity of >95%. Sorted lung activated ILC2s (aILC2s) were directly lysed to perform RNAseq analysis. Shown in **(B–D)** are the results from a 2 vs 2 comparison, with each sample being a lung aILC2 pool of 4 mice. **(B)** Volcano plot comparison depicting the expression of >13,000 transcripts detected in WT and ICAM-1^−/−^ lung aILC2s by the analysis. Differentially downregulated or upregulated genes (adj. p < 0.05 and 2-fold change cutoff) are presented in blue and yellow, respectively, with their respective numbers indicated in both upper corners of the plot. **(C, D)** Linear plot comparisons of WT and ICAM-1^−/−^ lung ILC2s depicting the expression of **(C)** cytokines and chemokines and **(D)** IL-10-producing ILC2 transcription factors based on a previous publication (26). Adjusted p-values: *Il10* = 0.048, *Cxcl15* = 0.002, *Il13* = 0.005, *Il9* = 0.024, *Il6* = 0.049, *Il5* = 0.004, *Csf2* = 0.049, *Cxcl2* = 0.004, *Cxcl3* = 0.048, *Il16* = 0.035, *Ccl6* = 0.015, *Ccl9* = 0.017, *Chil3* = 0.008, *Foxf1* = 0.049, *Id3* = 0.006, *Klf2* = 0.018, Prdm1 = 0.026, and Tcf7 = 0.010. **(E)** Cohorts of C57BL/6 (WT) and ICAM-1^−/−^ mice were challenged intranasally on days 1-3 with 0.5µg rmIL-33. On day 4, lungs were recovered and processed to single cell suspensions. After processing, lung cells were further stimulated with PMA, ionomycin and Brefeldin A for 4 h at 37°C prior staining for IL-10 intracellular cytokine marker. **(F)** Representative flow cytometry plots of intracellular IL-10 expression within total lung ILC2s of WT and LFA-1^−/−^ (gated as CD45^+^ Lin^−^ Thy1.2^+^ ST2^+^ CD127^+^) and **(G)** corresponding quantitation presented as mean frequency of IL-10–producing lung ILC2s in each cohort ± SEM. FMO: Full Minus One staining control. **(H)** BALB/cByJ mice were challenged intranasally on days 1 to 3 with 0.5 µg rmIL-33. On day 4, lungs were recovered, processed to single cell suspensions, and pulmonary activated ILC2s (gated as CD45^+^ Lin^−^ ST2^+^ CD127^+^) were FACS-sorted to a purity of >95%. Sorted lung aILC2s were then cultured *in vitro* (50 × 10^4^/ml) for 48 h with rmIL-2 (10 ng/ml), rmIL-7 (10 ng/ml) with or without anti-ICAM-1 (10 µg/ml). Culture supernatants were recovered for cytokine analysis by ELISA. **(I)** Levels of IL-5 and IL-13 in culture supernatants measured by ELISA. **(J)** Levels of IL-10 in culture supernatants measured by ELISA. Data are representative of 3 individual experiments with n = 5 except for RNAseq. *p < 0.05, **p < 0.005.

Based on our findings in **Figure 5**, we next performed exploratory transcriptomic analysis to assess the role of ICAM-1 and cell intrinsic factors that could potentially contribute to the modulation of AHR in response to IL-33. We therefore challenged cohorts of WT and ICAM-1^−/−^ mice with rmIL-33 on three consecutive days and performed RNA sequencing (RNAseq) analysis on FACS-sorted pulmonary ILC2s on day 4 (**Figure 6A**). Sort purities are shown in **Supplementary Figure 6A**, with used ILC2s >95% pure. The volcano plot presented in **Figure 6B** represents the >13,000 transcripts detected in the analysis according to their fold change (x axis, logFC) and statisticity (y axis, -log_10_ p-value). Our data suggest that a total of 455 transcripts were differentially regulated, with 50 downregulated and a remarkable 405 upregulated (**Figure 6B**). The top 50 genes upregulated and downregulated in the absence of ICAM-1 are found in **Supplementary Table 1**. Notably, we found both *Il9* and *Il13* were amongst the top 50 downregulated genes, confirming that loss of ICAM-1 affects main activation markers of ILC2s. To further assess the effect of ICAM-1 on cytokine and chemokine production, we applied a general cytokine/chemokine filter to our analysis (**Figure 6C**). In line with our previous findings, we found that ILC2 pro-inflammatory markers *Il5*, *Il9, Il13*, and *Csf2* were downregulated in ICAM-1^−/−^ ILC2s (**Figure 6C**). However remarkably, we observed a number of transcripts that were induced in the absence of ICAM-1, suggesting that lack of ICAM-1 may not only decrease pro-inflammatory cytokines in ILC2s. Interestingly, we found that *Il10* was the most upregulated interleukin in ILC2s lacking ICAM-1, and further found that *Il16*, *Ccl6*, *Ccl9* and *Cxcl15* were also upregulated (**Figure 6C**). IL-10-producing ILC2s (ILC2_10_) were described by several groups in multiple settings (26, 30, 31). Furthermore in the context of asthma, we recently found that IL-10-producing ILC2s significantly dampened AHR and lung inflammation, together providing evidence of their regulatory role in the inflamed lungs (32). In light of these findings, we found that signature transcription factors recently found to be induced in IL-10-producing ILC2s were similarly induced in ICAM-1^−/−^ ILC2s, namely *Id3*, *Foxf1*, *Klf2* (26), and *Prdm1* (32) (**Figure 6D**). We therefore next sought to confirm the expression of *Il10* at the protein level in activated lung ILC2s of WT and ICAM-1^−/−^ mice (**Figure 6E**). In line with our transcriptomic analysis, we found that ICAM-1^−/−^ activated lung ILC2s significantly upregulated by 3-fold the capacity of IL-10-production by pulmonary ILC2s upon *in vitro* PMA/ionomycin stimulation (**Figures 6F, G**). To further examine the effects of ICAM-1 on lung ILC2s, we next cultured WT activated ILC2s with a monoclonal anti-ICAM-1 blocking antibody *in vitro* and measured cytokine secretion in the culture supernatants after 48 h (**Figure 6H**). In confirmation of our previous findings, we first found that anti-ICAM-1 treatment downregulated IL-5 and IL-13 secretion by ILC2s (**Figure 6I**), while it remarkably upregulated the secretion of IL-10 (**Figure 6J**). Importantly, we made similar observations using ILC2s isolated from Rag^−/−^ mice that lack both T- and B-cells (**Supplementary Figures 6C, D**). Together, our preliminary results therefore confirm that ICAM-1 affects lung ILC2 effector functions in general. While it remarkably downregulated pro-inflammatory cytokines such as *Il5*, *Il9*, *Il13*, and *Csf2*, it however also notably upregulated other markers including *Il10* — recently described in IL-10-producing ILC2s — and opens new avenues for future investigations in the field.

## Discussion

In this study, we demonstrate that ILC2 expression of adhesion molecules LFA-1 and ICAM-1 play crucial roles in the development of IL-33-induced asthma symptoms, albeit at different levels. We found that expression by ILC2s of LFA-1 — but not ICAM-1 — is required for ILC2 trafficking to the lungs during inflammation. Additionally, blocking ICAM-1 on ILC2s reduced pro-inflammatory cytokine secretion and remarkably induced cytokines including IL-10. Together, our findings suggest that modulation of the LFA-1/ICAM-1 molecular pair on ILC2s may represent a promising and unappreciated novel therapeutic strategy regulating trafficking and cytokine production in the context of ILC2-dependent asthma.

LFA-1 is a crucial integrin involved in immune cell trafficking, as adhesion to ligand ICAM-1 facilitates firm adhesion to endothelium and prolonged contact with antigen presenting cells. Neutrophil expression of LFA-1 has been known for a long time to be involved in adherence and migration to inflamed tissues (27). This effect is however not restricted to innate cells, as it is now well appreciated that both T and B cells rely on LFA-1 for extravasation to lymph nodes and tissues (28, 29). Following IL-33 i.n. challenge, mice with constitutive lack of LFA-1 develop attenuated AHR associated with decreased accumulation of lung ILC2s as compared to controls. We found that both naïve and IL-33-activated lung ILC2s express high levels of LFA-1 subunits CD18 and CD11a, and our chimeric mice experiments importantly demonstrate that this expression of LFA-1 on ILC2s is specifically required for accumulation in the lungs. In confirmation of our findings, LFA-1 blockade using monoclonal antibodies against b2 integrin CD18 — known to dimerize with alpha subunits CD11a, CD11b, CD11c, or CD11d (12) — suggests that LFA-1 expression in mice is required for ILC2 accumulation in the lungs in the model of *Alternaria*-induced airway inflammation, although the localization of the targeted CD18 was not determined (10).

LFA-1 did not alter lung ILC2 numbers at steady state, nor did it affect ILC2 proliferation or survival in the lungs following IL-33 i.n. challenge, together suggesting that the difference in lung ILC2s observed is independent of ILC2 homeostasis. Little is known about the mechanisms underlying ILC2 infiltration in the airways upon inflammation. Our data however demonstrate that ILC2s are found in the peripheral blood following IL-33 i.n. stimulation. Interestingly, a previous report suggests that ILC2s in the lungs following *Alternaria* challenge are migrating from the bone marrow during inflammation (10). In support of this finding, we found that the bone marrow of mice challenged with IL-33 intranasally decreased compared to naïve mice, suggesting that ILC2s may egress from the bone marrow to the peripheral blood following intranasal stimulation with IL-33. However, further studies are required to better characterize this process, in particular how blood ILC2s contribute to lung pathogenesis. Interestingly, ILC2s found in the peripheral blood can also originate from the lungs themselves or other tissues such as the lamina propria as described previously (8). By focusing on the homing of activated ILC2s to the lungs, we found that the expression of LFA-1 by ILC2s was required for their infiltration to the lungs. In support of our findings, several reports show that adoptive transfer of activated lung ILC2s in Rag^−/−^ Il2rg^−/−^ alymphoid mice followed by three consecutive days of IL-33 intranasal challenge induces a potent ILC2-dependent airway inflammation and associated airway hyperreactivity (18–20). Our results therefore suggest that ILC2 infiltration to the lungs during inflammation — *via* ILC2 expression of LFA-1 — may contribute to the lung ILC2 pool and resulting ILC2-induced airway inflammation.

Although LFA-1 is widely known as a trafficking molecule, it was previously reported that LFA-1 on CD8^+^ T-cells is required for their retention in the mouse lungs (33). Our results do not exclude the contribution of LFA-1 in ILC2 retention in the lungs, as further studies are required to better characterize this process in the context of airway inflammation. Furthermore in the adipose tissue, it was recently demonstrated that a stromal cell niche expressing ICAM-1 is providing proliferation and activation signals to LFA-1-expressing ILC2s (34). Although lung adventitial stromal cells could provide such signals in the lungs (35), we did not observe a role for LFA-1 in ILC2 activation nor proliferation using our approach.

In the context of IL-33-induced asthma, we found that mice lacking ICAM-1 developed lower AHR and lung inflammation, and confirmed our results using a clinically proven anti-ICAM-1 blocking antibody in Rag^−/−^ mice (**Supplementary Figure 4**). Anti-ICAM-1-treated mice showed lower lung ILC2s, lower BAL eosinophilia associated with a decrease in ILC2-dependent IL-5 and IL-13 expression compared to controls. Although we found that ILC2s expressed ICAM-1, our chimeric mice experiment surprisingly revealed that ILC2 expression of ICAM-1 did not affect ILC2 accumulation in the lungs upon inflammation. However as reported previously, we found that the lack of ICAM-1 specifically on ILC2s resulted in a decrease of pro-inflammatory cytokines as well as proliferation, together further affecting lung inflammation and development of AHR (19). In support of our findings, Lei et al. in this study importantly show that ICAM-1 controls ILC2 activation, although they did not directly assess the requirement of ICAM-1 for ILC2 trafficking. Interestingly, we found that ICAM-1 was induced on ILC2s as early as 2 h following IL-33 challenge *in vitro*, as opposed to ICAM-2 which was highly expressed on ILC2s in both naïve and activated contexts. However, blocking ICAM-2 *in vivo* during IL-33-induced airway inflammation did not affect either the numbers of pulmonary ILC2s or BAL eosinophilia, suggesting that ICAM-2 — either on ILC2s or other cells — does not affect ILC2 accumulation in the lungs, at least sufficiently. In line with our observations, ICAM-1 on neutrophils was recently shown to affect neutrophil effector functions such as phagocytosis, but did not play a role in transmigration to inflamed tissues (36). In addition, human immature monocyte-derived dendritic cells treated with an ICAM-1 blocking antibody were shown to downregulate MHCI, MHCII, CD80, CD86 expression and cytokine secretion in response to LPS, suggesting that ICAM-1 on dendritic cells is required for efficient maturation and activation (37). Our results therefore rule out a role for ICAM-1 on ILC2s for their trafficking to the lungs but confirm ICAM-1 as a regulator of immune cell activation. It is important to note that soluble ICAM-1 (sICAM-1) in the sera is linked with asthma severity, suggesting that ICAM-1 may represent an adequate target for the treatment of the disease (38).

Our exploratory transcriptomic analysis confirmed previous reports that ICAM-1 controls pro-inflammatory cytokines (19). In addition, it showed that ICAM-1 may also negatively regulate the expression of chemokines/cytokines in ILC2s such as *Il16*, *Cxcl15*, *Ccl6*, and *Ccl9* in ILC2, which would all warrant further investigations. Remarkably, we found that *Il10* is the most upregulated interleukin in ICAM-1^−/−^ activated ILC2s, as we further confirmed our findings at the protein level as well as using a clinically proven anti-ICAM-1 blocking antibody *in vitro*. Interestingly, *Cxcl15* and *Chil3* were recently shown to be expressed by the pulmonary ILC2_10_ subset, as we further observed in ICAM-1^−/−^ ILC2s the expression of transcription factors upregulated in this population (26, 32). Although known producers of type 2 cytokines, it was recently demonstrated that ILC2s acquire the capacity to secrete cytokines not typically associated with their Th2 inflammatory phenotype. They were shown to produce IL-17 in a papain model of asthma (39), but more interestingly IL-10 and TGF-β in different models of asthma (26, 31, 40). IL-10 is a cytokine produced by T-regulatory cells, macrophages, and additional immune subtypes that is known for its anti-inflammatory properties (41). IL-10-producing ILC2s were described as anti-inflammatory, showing a specific transcription factors signature, but the exact mechanisms underlying their complex generation and control remains largely unknown (26). Interestingly, it was recently reported that intestinal ILC2s produce IL-10 at steady state, with IL-2, IL-4, IL-27, IL-10, and neuromedin U (NMU) enhancing IL-10 production in activated intestinal ILC2s (30). Importantly in the context of asthma, we recently found that IL-10–producing ILC2s significantly dampened AHR and lung inflammation, together providing evidence of their regulatory role in the inflamed lungs (32). Together, these findings suggest that IL-10-producing ILC2s are emerging as important players in the modulation of tissue inflammation, including the lungs. ICAM-1 may therefore represent a novel approach in the control of IL-10 production within ILC2s, as our findings pave the way for future investigations in the field. In support of our findings, it was previously reported that the engagement of ICAM-1 suppresses IL-10 production by human CD4^+^ T-cells (42). In this study, co-stimulation of ICAM-1 on activated CD4^+^ T-cells (but not CD8^+^ T-cells) *in vitro* increased IL-2 production, while remarkably inhibiting that of IL-10.

IL-10-producing ILC2s were described by several groups before and the production of IL-5 and IL-13 was always maintained in these cells (26, 30, 31, 40). Although the proportion of ILC2s producing IL-10 is low compared to that of IL-5 and IL-13, we believe that IL-10-producing ILC2s can contribute to the lung physiology. In support of our claim, a recent report showed that blocking TNF signaling in CD4^+^ T cells induced low but physiologically relevant levels of IL-10 as compared to Th1 signature cytokines, suggesting that TNF signaling similarly controls IL-10 secretion within CD4^+^ T-cells (43). Moreover, the induction of the same population of IL-10-producing ILC2s by Retinoic Acid at physiological levels made a similar significant impact on the phenotype (31). Although further studies are required to translate our findings to humans, it was recently reported that human ILC2s express ICAM-1 (19).

In conclusion, modulation of LFA-1 and/or ICAM-1 represents a promising and unappreciated novel therapeutic strategy regulating trafficking and cytokine production respectively in ILC2-dependent asthma.

## Data Availability Statement

The datasets generated for this study can be found in the GEO repository, GSE158652.

## Ethics Statement

The animal study was reviewed and approved by USC institutional Animal Care and Use Committee (IACUC).

## Author Contributions

BH designed, performed, and analyzed all experiments and wrote the manuscript. LG-T, PS-J, EH, DH, and JP performed experiments and animal husbandry for experiments. OA supervised, designed the experiments, interpreted the data, and finalized the manuscript. All authors contributed to the article and approved the submitted version.

## Funding

BH is supported by the Swiss National Science Foundation early postdoctoral mobility grant 181286. This article was financially supported by NIH Public Health Service grants R01 ES025786, R01 ES021801, R01 HL144790, R21 AI109059, and R01 HL151493 (OA).

## Conflict of Interest

The authors declare that the research was conducted in the absence of any commercial or financial relationships that could be constructed as a potential conflict of interest.

## Abbreviations

ILC2, group 2 innate lymphoid cell; IL, interleukin; TSLP, thymic stromal lymphopoietin; AHR, airway hyperreactivity; LFA-1, leukocyte factor associated antigen-1; ICAM, intercellular adhesion molecule; sICAM-1, soluble ICAM-1; WT, wild type; Rm, recombinant mouse; i.n., intranasal; aILC2, activated ILC2; nILC2, naïve ILC2; FACS, fluorescent-associated cell sorting; BAL, bronchoalveolar lavage; BM, bone marrow; RNAseq, RNA sequencing.
