# Supplementary material for: Distinct Roles of LFA-1 and ICAM-1 on ILC2s Control Lung Infiltration, Effector Functions, and Development of Airway Hyperreactivity
Source: Front Immunol. 2020 Oct 30;11:542818. doi: 10.3389/fimmu.2020.542818 (PMC7662114; doi:10.3389/fimmu.2020.542818)
Supplement: Supplementary file 1 [file DataSheet_1.pdf]

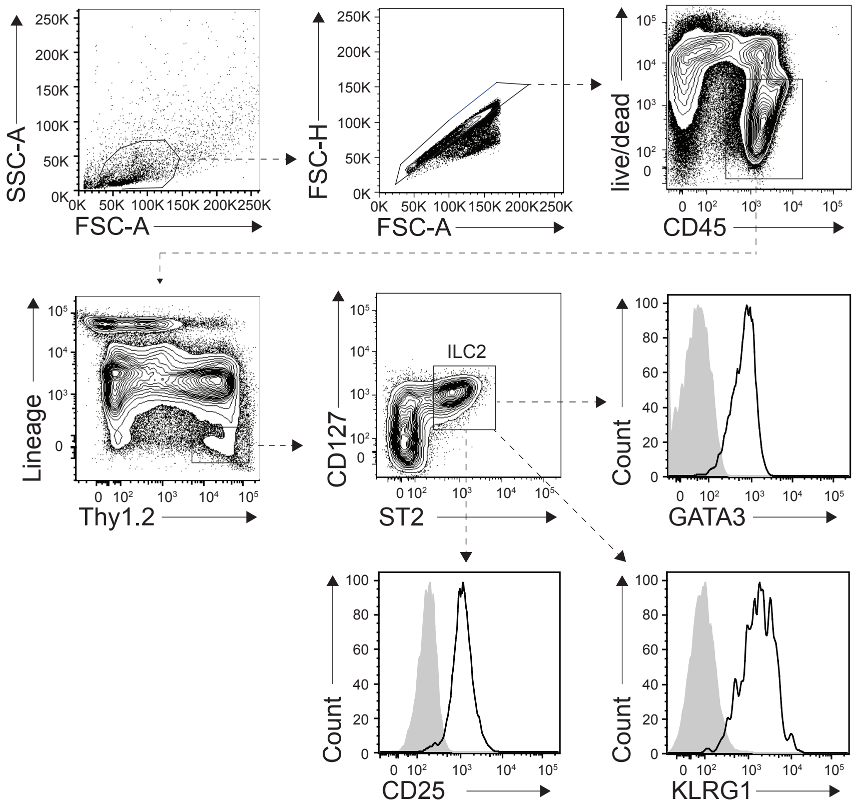

Supplementary Figure 1. Murine ILC2 gating strategy.

Representative flow cytometry plots for murine lung activated ILC2s. We first gated on lymphocytes and excluded cell doublets. We then gated on live CD45$^+$ cells and assessed ILC2s as Thy1.2$^+$, lineage (CD3ε, CD4, CD5, CD45R, Gr-1, CD11c, CD11b, Ter119, TCRγδ, TCRβ and FCεRIα) negative, ST2$^+$ and CD127$^+$ cells. Furthermore, ILC2s are CD25$^+$ and KLRG1$^+$, and express transcription factor GATA3. Gray solid histograms represent Full Minus One (FMO) staining controls.

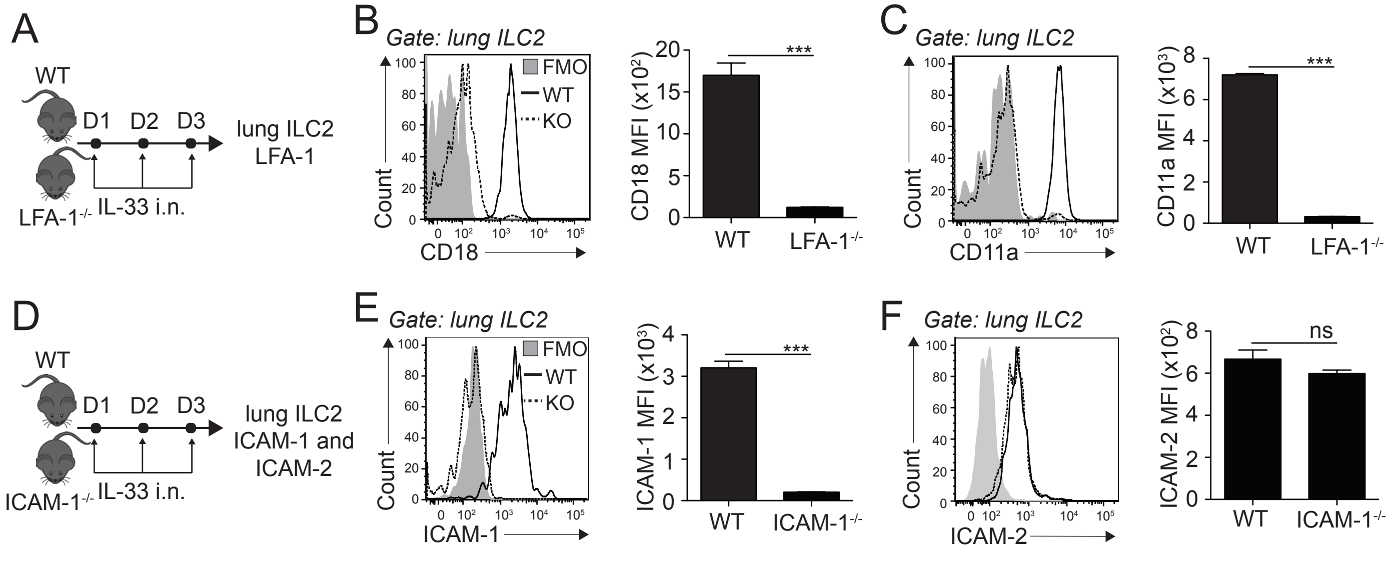

Supplementary Figure 2. LFA-1$^{-/-}$ and ICAM-1$^{-/-}$ mice models and expression of ICAM-2 in ICAM-1$^{-/-}$ mice

(A-C) Cohorts of C57BL/6 (WT) or LFA-1$^{-/-}$ mice were challenged intranasally on days 1-3 with 0.5μg rmIL-33 and on day 4 lungs were recovered, processed to single cell suspensions and the expression of CD18 and CD11a on pulmonary $CD45^+$ Lin^- $Thy1.2^+$ $ST2^+$ $CD127^+$ ILC2s was analyzed by flow cytometry.

(B and C) Representative flow cytometry plots of **(B)** CD18 and **(C)** CD11a expression on pulmonary ILC2s and corresponding quantitation presented as mean fluorescent intensity (MFI) +/- SEM. Black line: Corresponding marker, Solid Gray: Full Minus One (FMO) staining control.

(D-F) Cohorts of C57BL/6 (WT) or ICAM-1$^{-/-}$ mice were challenged intranasally on days 1-3 with 0.5μg rmIL-33 and on day 4 lungs were recovered, processed to single cell suspensions and the expression of ICAM-1 and ICAM-2 on pulmonary $CD45^+$ Lin^- $Thy1.2^+$ $ST2^+$ $CD127^+$ ILC2s was analyzed by flow cytometry.

(E and F) Representative flow cytometry plots of **(E)** ICAM-1 and **(F)** ICAM-2 expression on pulmonary ILC2s and corresponding quantitation presented as mean fluorescent intensity (MFI) +/- SEM. Black line: Corresponding marker, Solid Gray: Full Minus One (FMO) staining control.

Data are representative of 2 individual experiments with n=5. *** $p<0.001$, ns: non-significant.

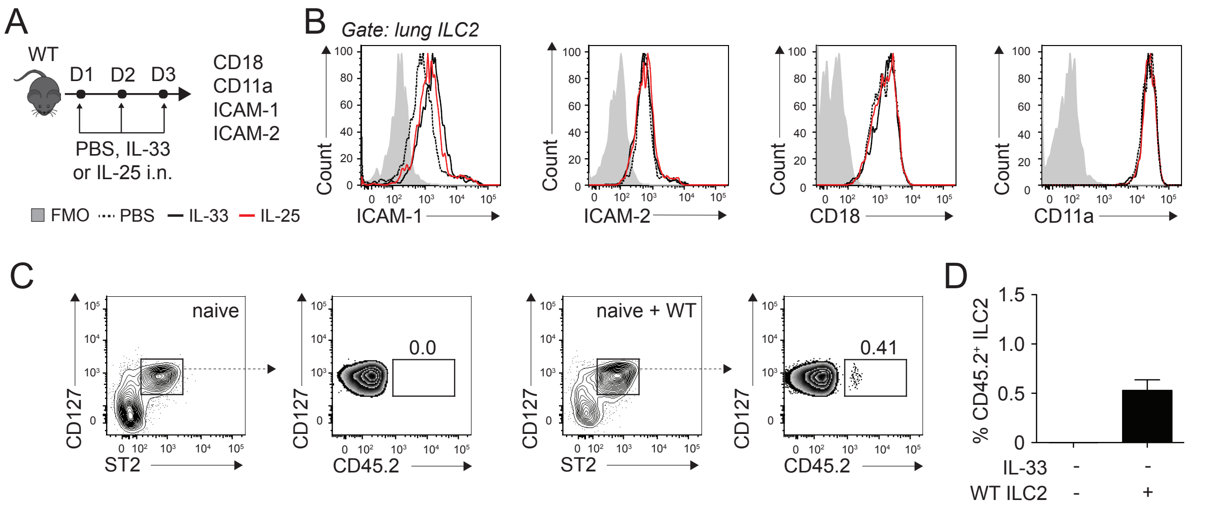

Supplementary Figure 3. Expression of adhesion molecules on lung ILC2s from C57BL/6 mice after IL-25 and IL-33 challenge and ILC2 homing to naïve lungs.

(A) Cohorts of C57BL/6 (WT) mice were challenged intranasally on days 1-3 with 0.5µg rmIL-33, 0.5µg rmIL-25 or PBS and on day 4 lungs were recovered, processed to single cell suspensions. The expression of ICAM-1, ICAM-2, CD18 and CD11a on pulmonary $CD45^+$ Lin^- $Thy1.2^+$ $ST2^+$ $CD127^+$ ILC2s was analyzed by flow cytometry.

(B) Representative flow cytometry plots of ICAM-1, ICAM-2, CD18 and CD11a expression on pulmonary ILC2s following challenge with rmIL-33, rmIL-25 or PBS. Black line: rmIL-33, red line: rmIL-25, dotted line: PBS, solid Gray: Full Minus One (FMO) staining control.

(C) A cohort of C57BL/6 (CD45.2) mice was challenged intranasally on days 1, 2 and 3 with 0.5µg rmIL-33. On day 4, lungs were recovered, processed to single cell suspensions and pulmonary activated ILC2s (aILC2)s were FACS-sorted to a purity of >95%. C57BL/6 CD45.1 naïve host mice were adoptively transferred or not with $10x10^3$ FACS-sorted $CD45.2^+$ aILC2 by tail-vein injection. On day 5, lungs were recovered, processed to single cell suspensions and analyzed by flow cytometry for the presence of pulmonary $CD45.2^+$ ILC2s.

(D) Representative flow cytometry plots of total pulmonary ILC2s in the corresponding cohorts and frequencies of $CD45.2^+$ adoptively transferred ILC2s within each population.

(G) Corresponding quantitation presented as the mean frequencies of $CD45.2^+$ within total lung ILC2s +/- SEM.

Data are representative of 2 individual experiments with n=5.

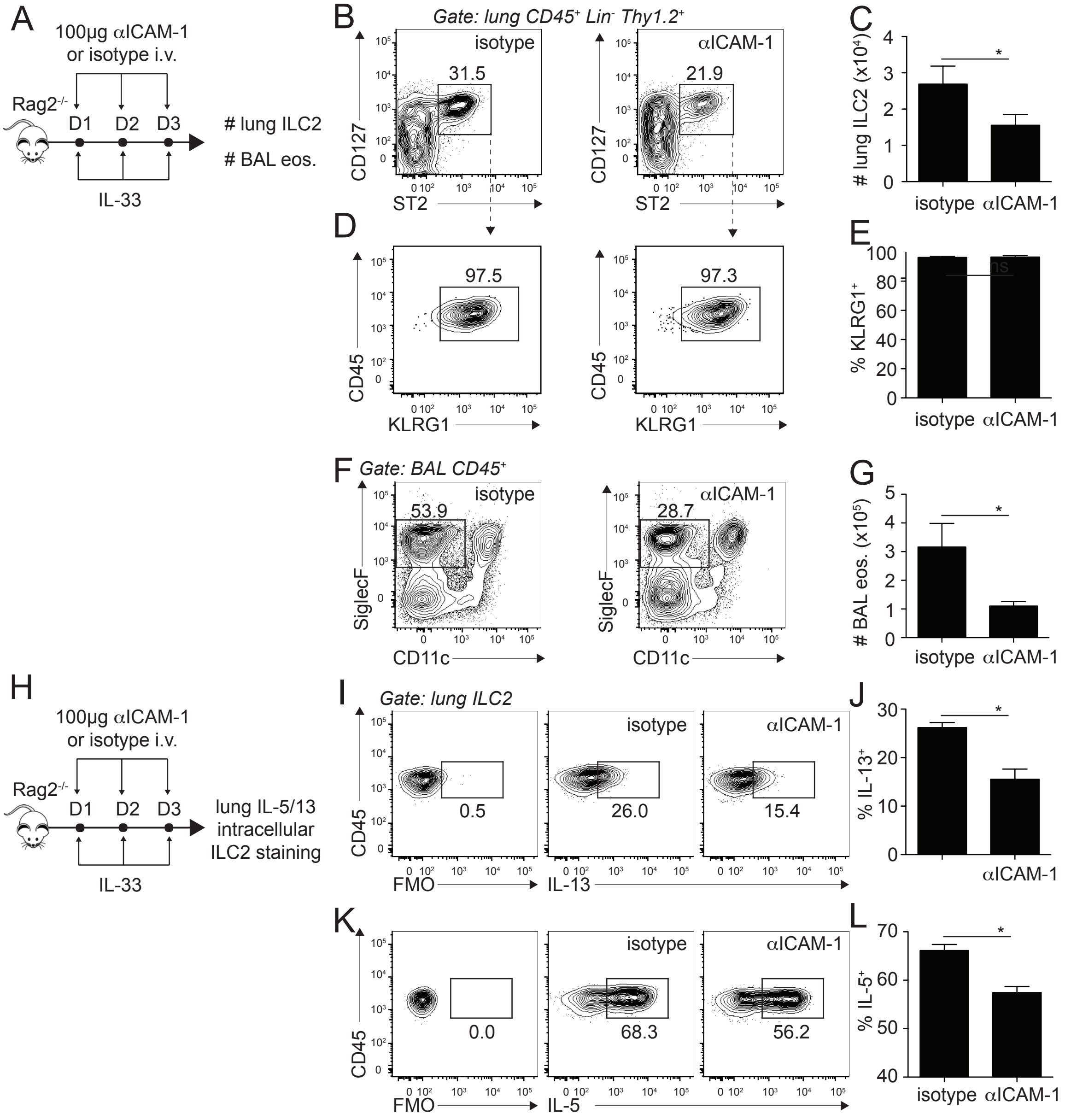

Supplementary Figure 4. Anti-ICAM-1 treated mice develop attenuated ILC2-dependent lung inflammation

(**A**) $Rag2^{-/-}$ mice received intravenous injections of 100μg αICAM-1 or isotype control and 0.5μg rmIL-33 intranasally on days 1-3. On day 4, bronchoalveolar (BAL) lavage was performed, prior collecting the lungs for each mouse. Lungs and BAL were processed to single cell suspensions and analyzed for ILC2s ($CD45^+$ Lin^- $Thy1.2^+$ $ST2^+$ $CD127^+$) and eosinophils by flow cytometry in lungs and BAL, respectively.

(B) Representative flow cytometry plots of lung ILC2s and **(C)** corresponding quantitation presented as the number of lung ILC2s +/- SEM.

(D) Representative flow cytometry plots of KLRG1 expression within lung ILC2s and **(E)** corresponding quantitation presented as the frequency of KLRG1-expressing ILC2s +/- SEM.

(F) Representative flow cytometry plots of BAL eosinophils and **(G)** corresponding quantitation presented as the number of BAL eosinophils +/- SEM.

Data are representative of 3 individual experiments with n=5. *p<0.05, ns: non-significant.

(H) Rag2$^{-/-}$ mice received intravenous injections of 100μg αICAM-1 or isotype control and 0.5μg rmIL-33 intranasally on days 1-3. On day 4, lungs were processed to single cell suspension and stimulated with PMA, ionomycin and Brefeldin A for 4 hours at 37C prior staining for intracellular cytokine markers within pulmonary ILC2s. Representative flow cytometry plots of IL-13 **(I)** and IL-5 **(K)** expression within lung ILC2 populations and corresponding quantitation **(J and L)** presented as mean frequency of cytokine expression within lung ILC2s +/- SEM.

Data are representative of 2 individual experiments with n=5. *p<0.05.

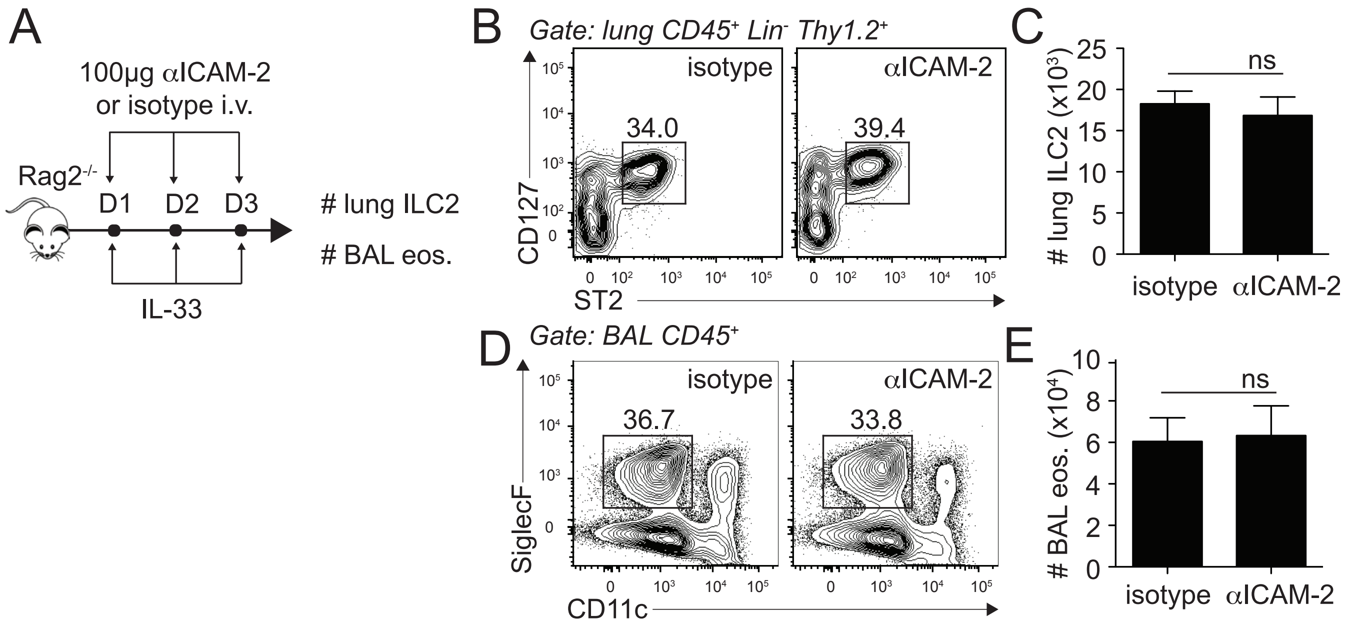

Supplementary Figure 5. Anti-ICAM-2 treated mice do not develop attenuated ILC2-dependent lung inflammation

(**A**) Rag2$^{-/-}$ mice received intravenous injections of 100µg αICAM-2 or isotype control and 0.5µg rmIL-33 intranasally on days 1-3. On day 4, bronchoalveolar (BAL) lavage was performed, prior collecting the lungs for each mouse. Lungs and BAL were processed to single cell suspensions and analyzed for ILC2s (CD45$^+$ Lin$^-$ Thy1.2$^+$ ST2$^+$ CD127$^+$) and eosinophils by flow cytometry in lungs and BAL, respectively.

(B) Representative flow cytometry plots of lung ILC2s and **(C)** corresponding quantitation presented as the number of lung ILC2s +/- SEM.

(D) Representative flow cytometry plots of BAL eosinophils and **(E)** corresponding quantitation presented as the number of BAL eosinophils +/- SEM.

Data are representative of 2 individual experiments with n=5. ns: non-significant.

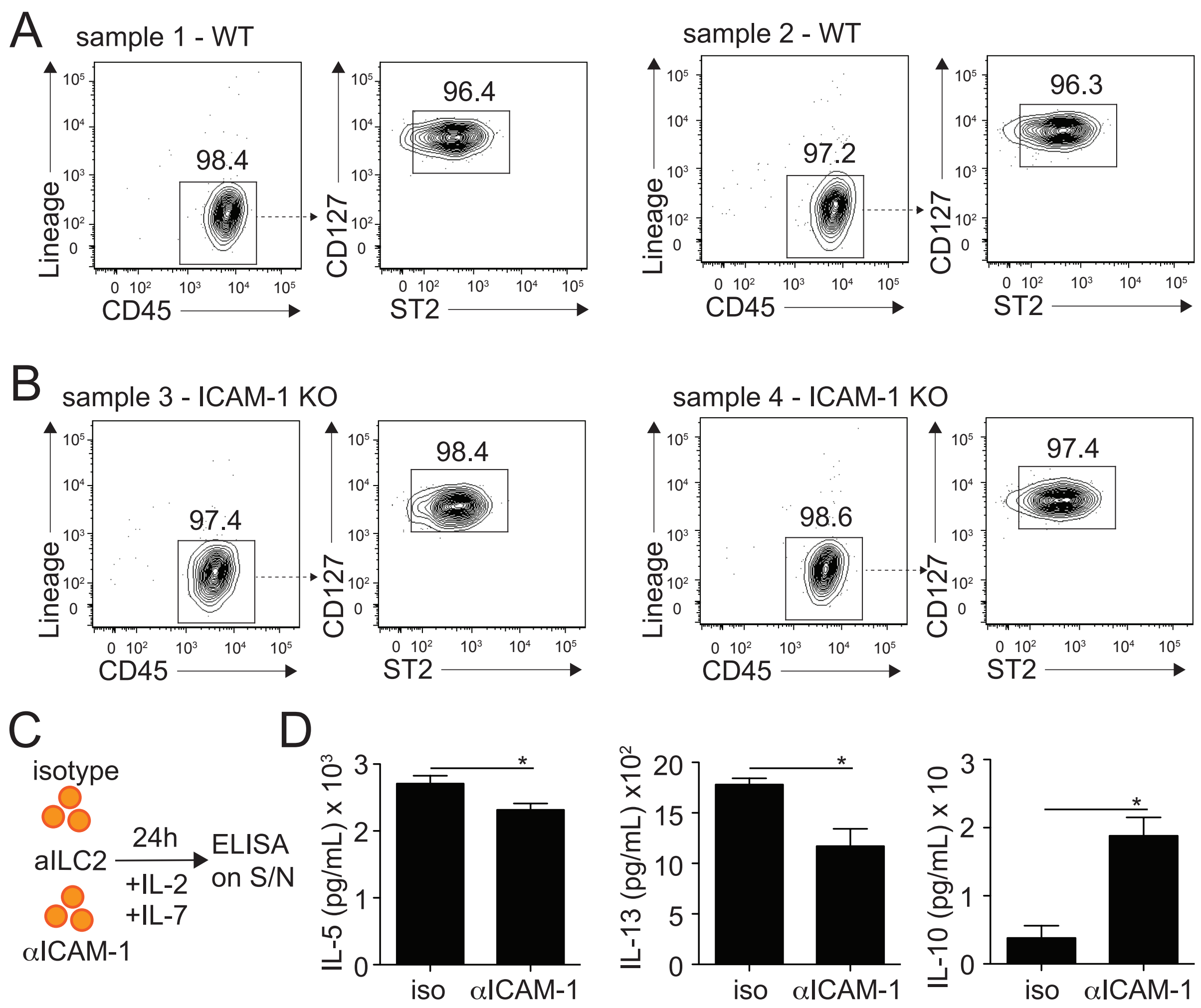

Supplementary Figure 6. RNAseq sample purities and role of ICAM-1 on in vitro cytokine production using ILC2s isolated from Rag$^{-/-}$ mice.

(A-B) Cohorts of C57BL/6 (WT) and ICAM-1$^{-/-}$ mice were challenged intranasally on days 1-3 with 0.5µg rmIL-33. On day 4, lungs were recovered, processed to single cell suspensions, and pulmonary activated ILC2s (gated as CD45^{+} Lin^{-} ST2^{+} CD127^{+}) were FACS-sorted to a purity of >95%. Sorted lung activated ILC2s (aILC2s) were directly lysed to perform RNAseq analysis. Each purified sample is a pool of 4 mice.

(C) Rag$^{-/-}$ mice were challenged intranasally on days 1-3 with 0.5µg rmIL-33. On day 4, lungs were recovered, processed to single cell suspensions, and pulmonary activated ILC2s (gated as CD45^{+} Lin^{-} ST2^{+} CD127^{+}) were FACS-sorted to a purity of >95%. Sorted lung aILC2s were then cultured *ex vivo* (50x10^{4}/mL) for 48h with rmIL-2 (10ng/mL), rmIL-7 (10ng/mL) with or without anti-ICAM-1 (10µg/mL). Culture supernatants were recovered for cytokine analysis by ELISA.

(D) Levels of IL-5, IL-13 and IL-10 in culture supernatants measured by ELISA.

Data presented in C and D are representative of 2 individual experiments with n=5. $^*p<0.05$.
